# Hypernatriämie und Polyurie bei einer Patientin mit Erstdiagnose einer sekundären akuten myeloischen Leukämie

**DOI:** 10.1007/s00108-022-01381-1

**Published:** 2022-07-15

**Authors:** E. Aydilek, K. Jäger, K. Xhaxho, R. Akdas, M. Wallbach, G. Wulf, N. Brökers

**Affiliations:** 1 Klinik für Hämatologie und medizinische Onkologie, Universitätsklinikum Göttingen, Robert-Koch-Str. 40, 37075 Göttingen, Deutschland; 2 Klinik für Gastroenterologie und intestinale Onkologie, Universitätsklinikum Göttingen, Göttingen, Deutschland; 3 Klinik für Nephrologie und Rheumatologie, Universitätsklinikum Göttingen, Göttingen, Deutschland

**Keywords:** Sekundäre AML, Monosomie 7, Translokation 3, Zentraler Diabetes insipidus, Paraneoplastisches Syndrom, Secondary AML, Monosomy 7, Translocation 3, Central diabetes insipidus, Paraneoplastic syndrome

## Abstract

Dargestellt wird der Krankheitsverlauf einer 66-jährigen Patientin mit der Erstdiagnose einer akuten myeloischen Leukämie. Als paraneoplastisches Syndrom zeigten sich eine Hypernatriämie sowie Polyurie infolge eines zentralen Diabetes insipidus (CDI), welcher mittels Desmopressingabe kontrolliert werden konnte. Nach Einleitung einer Induktionstherapie kam es im Verlauf nach Beendigung der Desmopressintherapie noch vor Anstieg des Blastenanteils zu einer erneuten Hypernatriämie und Polyurie als Hinweis auf eine primäre Refraktärität.

## Abstract (en)

A 66-year-old female patient with the initial diagnosis of acute myeloid leukemia is reported. Paraneoplastic syndrome manifested as hypernatremia due to central diabetes insipidus (CDI), which could be controlled with the administration of desmopressin. After initiation of the induction therapy, the required desmopressin administration could be reduced and terminated. In the further course, the early increasing polyuria and hypernatremia indicated the primary refractory acute myeloid leukemia.

## Anamnese

Die Übernahme der 66-jährigen Patientin erfolgte mit der Verdachtsdiagnose einer akuten myeloischen Leukämie (AML) aus einem externen Krankenhaus, wo sie sich mit zunehmender Erschöpfung vorstellte. In der externen laborchemischen Diagnostik waren neben einer akuten Nierenschädigung (AKI) 4 % Blasten im peripheren Blut nachweisbar. Fremdanamnestisch war zu eruieren, dass die Patientin noch einige Tage zuvor bei guter Belastbarkeit vollständig selbstversorgend gewesen sei. Ein gesteigertes Durstgefühl und eine gesteigerte Trinkmenge wurden nicht angegeben.

2017 erkrankte die Patientin an einem invasiven Mammakarzinom, war nach Durchführung einer neoadjuvanten Therapie und anschließend brusterhaltenden Therapie rezidivfrei.

## Diagnostik

### Klinischer Befund

Bei Aufnahme präsentierte sich die Patientin in reduziertem und exsikkiertem Allgemeinzustand. Die Patientin war anfangs zu allen Qualitäten orientiert und ohne fokal-neurologisches Defizit. Der Verdacht eines Herpes labialis wurde geäußert, die sonstige klinische Untersuchung war ohne wegweisenden Befund. Auffällig waren im weiteren Verlauf eine progrediente Polyurie (bis zu 6 l/Tag) sowie eine zunehmende Desorientiertheit mit sensorischer Aphasie und Gangataxie.

### Labordiagnostik

**Figure 1:**
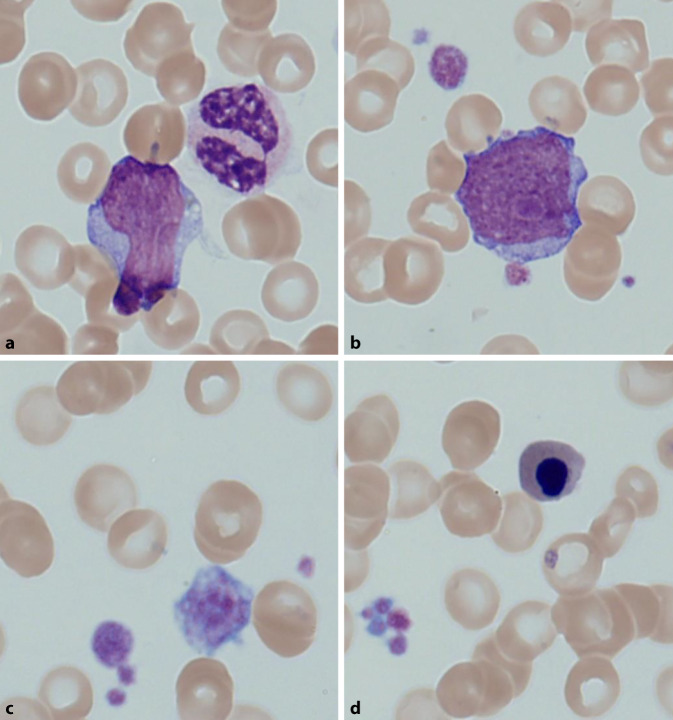

**Figure 2:**
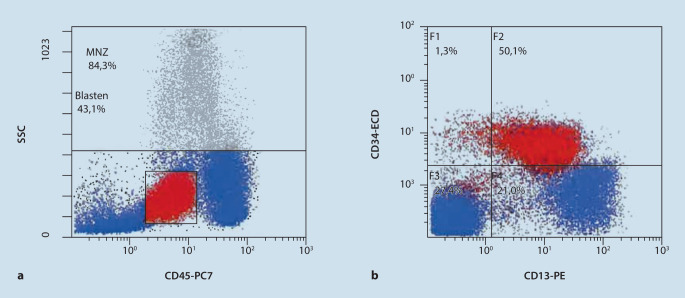

**Figure 3:**
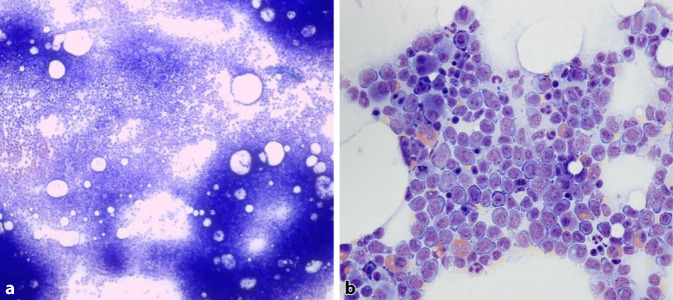

Das Differenzialblutbild bestätigte die vorbeschriebene Blastenausschwemmung (37 %). Daneben fanden sich Dysplasiezeichen der Thrombozyten sowie der Granulozyten (siehe Abb. 1 und Tab. 1). Aufgrund des durchflusszytometrischen Phänotyps (Expression von u. a. CD13+, CD34+; siehe Abb. 2) sowie des Blastenanteils wurde die Diagnose einer AML gestellt. In der Knochenmarksdiagnostik war neben der Blastenvermehrung eine multilineare Dysplasie nachweisbar, die Megakaryopoese, Erythropoese und Granulozyten betraf (jeweils > 50 %), sodass die Subgruppe einer AML mit myelodysplasieassoziierten Veränderungen (AML-MRC) diagnostiziert werden konnte (siehe Abb. 3). Die weiteren Untersuchungen erbrachten zytogenetisch den Nachweis einer Monosomie 7 und Translokation t(3;3) und molekulargenetisch Mutationen in den Genen *NRAS* und *PTPN11*. In der Zusammenschau lag gemäß der Klassifikation des European LeukemiaNet (ELN) eine ungünstige Prognose vor.

**Table 1:** 

Hämatologische Diagnostik			
Hämoglobin	11,5–15,0	g/dl	9,8
Hämatokrit	35–46	%	29,7
Erythrozyten	3,9–5,1	10^6^/µl	2,90
MCV	81–95	fl	102
MCH	26,0–32,0	pg	33,8
MCHC	32,0–36,0	g/dl	33,0
Thrombozyten	150–350	10^3^/µl	307
Leukozyten	4,0–11,0	10^3^/µl	15,70
*Differenzialblutbild*			
Blasten (nicht klass.)	–	%	37
Myelozyten	–	%	2
Metamyelozyten	–	%	1
Stabkernige	≤ 8	%	3
Segmentkernige	40–76	%	8
Lymphozyten	20–45	%	26
Monozyten	3–13	%	19
Eosinophile	≤ 8	%	3

In der weiteren laborchemischen Diagnostik (Tab. 2) präsentierte sich eine AKI im Stadium II. Parameter einer Proliferation waren erhöht (LDH, Cystatin C). Klare Hinweise auf eine Tumorlyse ergaben sich bei normwertigem Phosphor und leichter Hyperurikämie laborchemisch nicht. Auffällig war zudem eine Hypernatriämie, begleitet von einer erhöhten Serumosmolalität (319 mosm/kg) bei erniedrigter Urinosmolalität (161 mosm/kg). Aufgrund der laufenden Therapie mit Aciclovir bei V. a. Herpes labialis bestand zunächst die Annahme einer AKI in polyurischer Phase. Bei Ausbleiben einer Besserung und Zunahme der Polyurie und Therapieansprechen auf Desmopressin wurde die Diagnose eines Diabetes insipidus centralis (CDI) gestellt.

**Table 2:** 

Laborchemische Diagnostik			
Natrium	136–145	mmol/l	154
Kalium	3,5–4,6	mmol/l	3,8
Kreatinin	0,50–1,00	mg/dl	1,61
Harnsäure	2,6–6,0	mg/dl	7,5
Laktat-Dehydrogenase	125–250	U/l	344
Osmolalität im Serum	275–300	mosm/kg	319
Natrium im Spontanurin	–	mmol/l	61
Osmolalität im Urin	50–1200	mosm/kg	161
Cystatin C	0,40–0,99	mg/l	2,34
Phosphor, anorg.	0,74–1,52	mmol/l	0,89

### Ergänzende Diagnostik

Mittels nativer Computertomographie des Schädels konnten Akutpathologien ausgeschlossen werden. Eine zerebrale MRT-Diagnostik war aufgrund des klinischen Zustands nicht durchführbar. Eine Computertomographie des gesamten Integuments war ohne Hinweis auf ein Rezidiv des behandelten Mammakarzinoms. Die Liquoranalyse erbrachte weder den Nachweis einer Entzündung noch von Tumorzellen.

## Diagnose

- Sekundäre akute myeloische Leukämie vom Typ AML-MRC mit ungünstiger Prognose nach ELN und Entwicklung eines Diabetes insipidus centralis als paraneoplastisches Syndrom.

## Therapie und Verlauf

**Figure 4:**
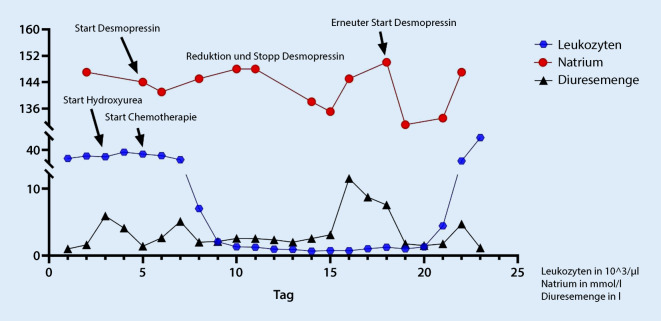

Aufgrund der zunehmenden Verwirrtheit war die Überwachung und Weiterbehandlung auf einer Intermediate-Care-Station notwendig. Mit dem Ziel einer Stabilisierung wurde zunächst eine zytoreduktive Therapie mit Hydroxyurea begonnen, jedoch ohne dass eine signifikante Verbesserung eintrat. Eine Induktionstherapie nach dem 7 + 3-Schema (Cytarabin und Doxorubicin) wurde eingeleitet. Begleitend dazu wurde regelmäßig – unter engmaschiger Kontrolle des Serumnatriums bzw. des Volumenstatus einschließlich Urinausscheidung – die Gabe von Desmopressin durchgeführt. Die Dosierung konnte während der Phase der Aplasie deeskaliert und schließlich pausiert werden. Im Verlauf kam es zu einem rasanten Anstieg der Leukozyten mit einem Blastenanteil von ca. 40 %, sodass eine refraktäre Leukämie vorlag. Bereits 5 Tage vor Nachweis des Blastenschubs kam es zu einer Zunahme der Polyurie bzw. Hypernatriämie, sodass die Desmopressintherapie wieder aufgenommen werden musste. Die Patientin verstarb an den Folgen der refraktären AML (Abb. 4).

## Diskussion

Als paraneoplastisches Syndrom (Paraneoplasie) werden Funktionsstörungen bzw. Symptome benannt, die im Rahmen einer Krebserkrankung auftreten können. Eine Paraneoplasie kann in ca. 7 % der Patient*innen mit einer Krebserkrankung auftreten und geht häufig der eigentlichen Diagnose voraus [1]. Neben neurologischen, dermatologischen und rheumatologischen Symptomen können endokrinologische Veränderungen auftreten. Das klinische Bild einer AML ist meistens heterogen und hervorgerufen durch die hämatopoetische Insuffizienz. Ein paraneoplastisches Syndrom bei dieser Erkrankung ist grundsätzlich selten, manifestiert sich häufig dermal, kann der eigentlichen Diagnosestellung vorausgehen und schwere Komplikationen hervorrufen.

In der präsentierten Kasuistik manifestierten sich eine zunehmende Hypernatriämie sowie Polyurie, begleitet von schweren neurologischen Funktionseinschränkungen. Im Rahmen der Abklärung wurde die Diagnose eines CDI gestellt, der eine seltene Paraneoplasie der AML darstellt und typischerweise mit einer Monosomie 7 bzw. Translokation t(3;3) einhergeht [2]. Der zugrunde liegende pathophysiologische Mechanismus ist hierbei nicht abschließend geklärt. Diskutiert wird z. B. eine leukämische Infiltration der Neurohypophyse. Eine komplette Remission wird trotz intensiver Therapie selten erreicht und die Prognose ist insgesamt schlecht. Als Einzelfallberichte sind Langzeitremissionen nach einer allogenen Stammzelltransplantation erreicht worden [3].

Im hier beschriebenen Fall wies nach Beendigung der Desmopressingabe eine erneute Hypernatriämie mit Zunahme der Polyurie noch vor Nachweis einer erneuten peripheren Blastenausschwemmung auf eine primäre Refraktärität der Erkrankung hin.

## Fazit für die Praxis

- Die Symptome einer AML sind unspezifisch und nicht immer durch die hämatopoetische Insuffizienz hervorgerufen.
- Das Wiederauftreten paraneoplastischer Syndrome kann ein Rezidiv anzeigen.
- Die Prognose einer AML bei o. g. Chromosomenkonstellation ist insgesamt schlecht.
